# Safety and Usefulness of Intracoronary Acetylcholine 200 μg Into the Left Coronary Artery as Vasoreactivity Testing: Comparisons With Intracoronary Acetylcholine Maximum 100 μg

**DOI:** 10.1002/clc.70001

**Published:** 2024-10-02

**Authors:** Shozo Sueda, Yutaka Hayashi, Hiroki Ono, Tomoki Sakaue, Shuntaro Ikeda

**Affiliations:** ^1^ Department of Cardiology Minami Matsuyama Hospital Matsuyama Japan; ^2^ Department of Cardiology Ehime Prefectural Niihama Hospital Niihama Japan; ^3^ Department of Cardiology Ehime University Graduate School of Medicine Matsuyama Japan

**Keywords:** acetylcholine 100 μg, acetylcholine 200 μg, left coronary artery, safety, usefulness, vasoreactivity testing

## Abstract

**Objectives:**

We retrospectively analyzed the usefulness and safety of intracoronary acetylcholine (ACh) 200 μg into the left coronary artery (LCA) as vasoreactivity testing compared with intracoronary ACh 100 μg.

**Methods:**

We recruited 1433 patients who had angina‐like chest pain and intracoronary ACh testing in the LCA, including 1234 patients with a maximum ACh 100 μg and 199 patients with a maximum ACh 200 μg. ACh was injected in incremental doses of 20/50/100/200 μg into the LCA. Positive spasm was defined as ≥ 90% stenosis, usual chest pain, and ischemic electrocardiogram (ECG) changes.

**Results:**

The incidence of coronary constriction ≥ 90%, usual chest pain, and ischemic ECG changes with a maximum ACh of 100 μg was markedly higher than that with a maximum ACh of 200 μg. The frequency of unusual chest pain in patients with a maximum ACh of 200 μg was higher than that in those with a maximum ACh of 100 μg (13% vs. 3%, *p* < 0.001). In patients with rest angina, positive spasm of maximum ACh 100 μg was significantly higher than that of maximum ACh 200 μg, whereas there was no difference regarding positive spasm in patients with atypical chest pain between the two ACh doses. Major complications (1.38% vs. 1.51%, *p* = 0.8565) and the occurrence of paroxysmal atrial fibrillation (1.81% vs. 2.63%, *p* = 0.6307) during ACh testing in the LCA were not different between the two maximum ACH doses.

**Conclusions:**

Intracoronary ACh 200 μg into the LCA is clinically useful and safe for vasoreactivity testing when intracoronary ACh 100 μg dose not provoke spasms.

## Graphical abstract

Comparisons of the vasoreactivity testing results after propensity score matching.

After propensity score matching, there are no differences regarding positive spasms, usual chest pain, or major complications between patients with a maximum ACh of 200 μg and those with a maximum ACh of 100 μg before August 2012. However, the frequency of unusual chest pain in patients with a maximum ACh of 200 μg is markedly higher than in those with a maximum ACh of 100 μg before August 2012 after propensity score matching.

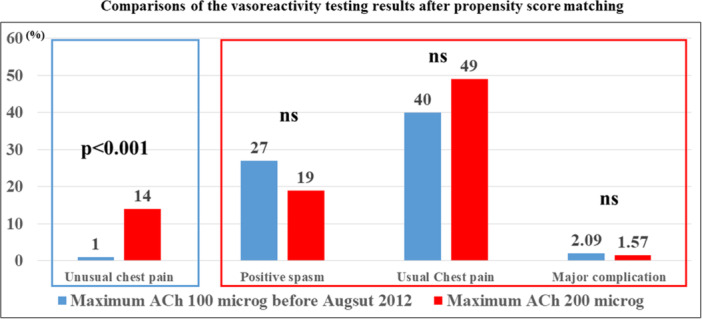

## 1 Introduction

Yasue et al. reported the clinical usefulness and safety of intracoronary acetylcholine (ACh) as a spasm provocation test in 1986, when cardiologists reproduced ST segment elevation in patients with variant angina by ACh vasoreactivity testing [1]. Okumura et al. reported the sensitivity and specificity of intracoronary ACh testing (maximum ACh 50 μg in the right coronary artery [RCA] and maximum ACh 100 μg in the left coronary artery [LCA]) as 89% or 99% [2]. The sensitivity of these ACh tests in the LCA and RCA was 88% (38/43) and 90% (44/49), respectively. However, they could not reproduce provoked spasm by intracoronary ACh testing in 7 of 70 variant angina patients. Even in patients with variant angina who had high disease activity, intracoronary injection of ACh could not obtain perfect results of reproducible spasm. Western cardiologists such as Ong et al. have employed the intracoronary ACh 200 μg into the LCA since December 2007 [3]. They employed the same protocol of the ENCORE I (Evaluation of Nifedipine and Cerivastatin on the Recovery of Endothelial Function) study [4]. We have employed the intracoronary ACh 200 μg more than 100 μg in the LCA since 2012 [5]. However, the Japanese Circulation Society (JCS) guidelines recommend a maximum ACh dose of 100 μg into the LCA [6].

In this article, we retrospectively analyzed provoked coronary constriction (≥ 90%), usual chest pain, ischemic electrocardiogram (ECG) changes, and complications during ACh maximum 100 or 200 μg spasm provocation testing in the LCA.

## 2 Methods

### 2.1 Study Patients

**Figure 1:**
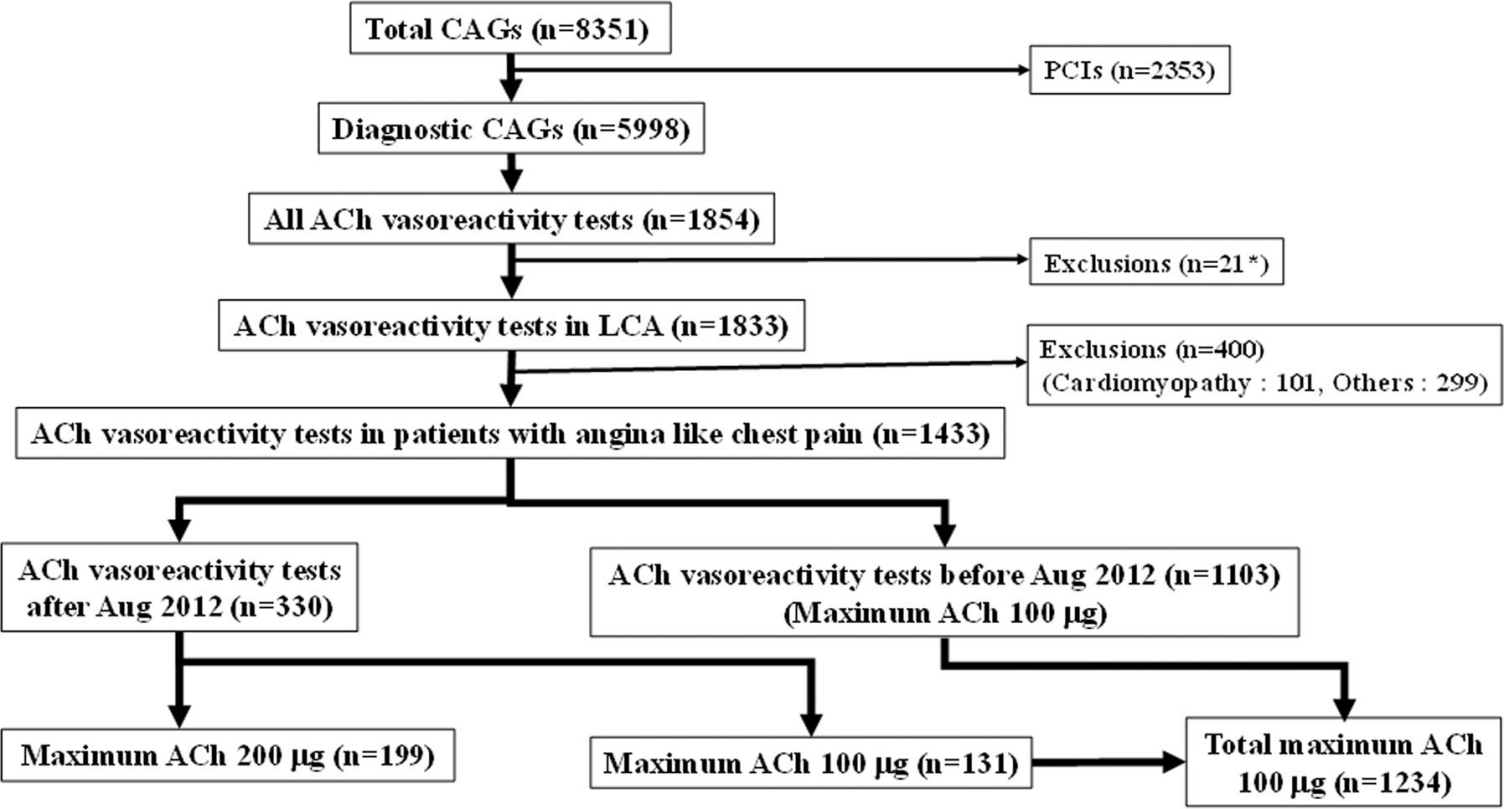
Flowchart of this study. *We could not perform ACh testing in the LCA. ACh, acetylcholine; CAG, coronary angiography; LCA, left coronary artery.

From January 1991 to February 2019, we performed a total of 8351 coronary angiography procedures, including 2353 percutaneous coronary interventions (PCIs) and 5998 diagnostic and follow‐up cardiac catheterization procedures as shown in Figure 1. Simultaneously, we performed spasm provocation tests of ACh in 1854 patients. As shown in Table 1 and Figure 1, we recruited 1433 patients who had angina like chest pain and had intracoronary ACh testing in the LCA, including 1234 patients (1103 patients before August 2012 and 131 patients after August 2012) with a maximum ACH of 100 μg and 199 patients with a maximum ACh of 200 μg. We have employed intracoronary ACh 200 μg more than 100 μg since August 2012. Subjects were excluded, and the provocation test was not performed if patients had triple‐vessel disease, two‐vessel disease with total occlusion, heart failure (New York Heart Association functional class III or IV), renal failure (creatinine > 2.0 mg/dL), spontaneous spasm or if isosorbide dinitrate was initially used to relieve spasm in the coronary artery tested.

**Table 1:** Comparisons of clinical characteristic of patients who underwent intracoronary acetylcholine testing in the left coronary artery.

	Total ACh tests	(A) Total maximum ACh 100 μg	*p* value (A vs. B)	(B) Total maximum ACh 200 μg	*p* value (B vs. C)	(C) Maximum ACh 100 μg before August 2012	Maximum ACh 100 μg after August 2012
Number of patients	1433	1234		199		1103	131
Female	389 (27%)	330 (27%)	0.392	59 (30%)	0.733	312 (28%)	18 (14%)***
Age (years)	65 ± 11	65 ± 10	0.445	66 ± 11	0.585	64 ± 10	67 ± 11
Organic stenosis in the LCA (> 75%)	287 (20%)	278 (23%)	< 0.001	9 (5%)	< 0.001	268 (24%)	10 (8%)***
Hypertension	683 (48%)	557 (45%)	< 0.001	126 (63%)	< 0.001	491 (45%)	66 (50%)
Dyslipidemia	726 (51%)	609 (49%)	0.014	117 (59%)	0.002	518 (47%)	91 (69%)***
Diabetes mellitus	375 (26%)	315 (26%)	0.168	60 (30%)	0.160	279 (26%)	36 (27%)
History of smoking	995 (69%)	867 (70%)	0.091	128 (64%)	0.186	762 (69%)	105 (80%)**
Clinical diagnosis							
Ischemic heart disease	1267 (88%)	1088 (88%)	0.466	179 (90%)	0.295	960 (87%)	128 (98%)***
Rest angina	510 (36%)	428 (35%)	0.074	82 (41%)	0.052	374 (34%)	54 (41%)
Effort angina	157 (11%)	139 (11%)	0.352	18 (9%)	0.458	124 (11%)	15 (11%)
Rest and effort angina	130 (9%)	117 (9%)	0.178	13 (7%)	0.275	101 (9%)	16 (12%)
Myocardial infraction	203 (14%)	194 (16%)	< 0.001	9 (5%)	< 0.001	190 (17%)	4 (3%)***
Previous PCI	267 (19%)	210 (17%)	< 0.001	57 (29%)	< 0.001	171 (16%)	39 (30%)***
Non‐ischemic heart disease							
Atypical chest pain	166 (12%)	146 (12%)	0.466	20 (10%)	0.295	143 (13%)	3 (2%)***
Medications at baseline							
Calcium channel blockers	800 (56%)	650 (53%)	< 0.001	150 (75%)	< 0.001	563 (51%)	87 (66%)***
Nitrates or nicorandils	617 (43%)	535 (43%)	0.569	82 (41%)	0.394	484 (44%)	51 (39%)
ACEI or ARBs	281 (20%)	223 (18%)	< 0.001	58 (29%)	< 0.001	194 (18%)	29 (22%)
Beta‐blockers	151 (11%)	114 (9%)	< 0.001	37 (19%)	< 0.001	99 (9%)	15 (11%)
Statins	311 (22%)	235 (19%)	< 0.001	76 (38%)	< 0.001	183 (17%)	52 (40%)***
Anti‐platelets	524 (37%)	447 (36%)	0.502	77 (39%)	0.577	402 (36%)	45 (34%)

Abbreviations: ACEI, angiotensin converting enzyme inhibitor; ACh, acetylcholine; ARB, angiotensin receptor blocker; PCI, percutaneous coronary intervention; RCA, right coronary artery.

** *p* < 0.01

*** *p* < 0.001 versus maximum ACh 100 μg before August 2012.

### 2.2 The Definition of Positive Spasm, Provoked Spasm Phenotypes, and Major Complications During Intracoronary ACh Testing

We defined positive spasm as ≥ 90% transient stenosis associated with the usual chest symptoms and ischemic ECG changes and defined negative spasm as < 90% transient stenosis associated with neither usual chest pain nor ischemic ECG changes. The remaining responses were defined as unclassified results. Focal spasm was defined as a total or subtotal obstruction within the borders of one isolated coronary segment, and diffuse spasm was defined as severe diffuse constriction (90% stenosis) observed in ≥ 2 adjacent coronary segments. The degree of ST‐segment depression was measured at 80 ms after the J point. We considered a result to be positive when at least one of the following ischemic ECG changes was demonstrated during and/or after the ACh test: (1) ST‐segment elevation of ≥ 0.1 mV in at least two contiguous leads; (2) ST‐segment depression of 0.1 mV in at least two contiguous leads. We also considered a negative U wave as a positive ischemic ECG change. We defined major complications during pharmacological testing as death, acute myocardial infarction, ventricular fibrillation, sustained or nonsustained ventricular tachycardia, shock, hypotension (< 60 mmHg), or cardiac tamponade.

### 2.3 Spasm Provocation Test

All drugs except for nitroglycerine were discontinued for ≥ 24 h before the study, and nitroglycerine was also discontinued ≥ 4 h before the study. Cardiac catheterization was performed from 9:00 a.m. to 4:00 p.m. in the fasting state. After control coronary arteriograms of the LCA and the RCA were obtained by injection of 8−10 mL of contrast medium, a temporary pacemaker was inserted into the right ventricle of each ACh testing patient, and the pacing rate was set at 40−45 beats/min.

Provocation of coronary artery spasm was performed with an intracoronary injection of ACh, as previously reported [7, 8]. ACh chloride (Neucholin‐A, 30 mg/2 mL; Zeria Seiyaku, Tokyo, Japan) was injected in incremental doses of 20, 50, and 80 μg into the RCA and of 20, 50, 100, and 200 μg into the LCA over 20 s with at least a 3 min interval between each injection. Coronary arteriography was performed when ST‐segment changes and/or chest pain occurred or 1−2 min after the completion of each injection. When an induced coronary spasm did not resolve spontaneously within 3 min after the completion of ACh injections or when hemodynamic instability occurred as the result of coronary spasm, 2.5−5.0 mg of nitrate was injected into the involved vessel. A standard 12‐lead ECG was recorded every 30 s. We used the ECG findings when ACh, saline, and contrast medium were not injected into the responsible vessels for at least 60 s. After the spasm provocation tests were completed, an intracoronary injection of 5.0 mg isosorbide dinitrate was administered, and coronary arteriography was then performed into multiple projections.

During the study, arterial blood pressure and ECG were continuously monitored on an oscilloscope by Nihon‐Kohden polygraphy (Tokyo, Japan). In the present study, coronary arteriograms were analyzed separately by two independent observers. The percent luminal diameter narrowing of coronary arteries was measured using an automatic edge‐counter detection computer analysis system. The size of the coronary catheter was used to calibrate the images in millimeters, and the measurement was performed in the same projection of coronary angiography at each stage. Patients with catheter‐induced spasms were excluded from this study. Significant organic stenosis was defined as > 75% luminal narrowing, and proximal spasm, mid spasm, and distal spasm were defined as sites of segment 5/6 or 11, segment 7, or segment 8/9/10 or 12/13/14/15, respectively, according to the American College of Cardiology/American Heart Association classification [9].

The study protocol complied with the Declaration of Helsinki. Written informed consent was obtained from all patients before performing the pharmacological spasm provocation tests, and the protocol of this study was in agreements with the guidelines of the ethical committee at our institution.

### 2.4 Statistical Analysis

Data analysis was carried out with SPSS (version 22.0, IBM Japan Ltd., Tokyo, Japan). All data are presented as the mean ± 1 standard deviation (SD). Clinical characteristic, including coronary risk factors, provoked spasm incidence, occurrence of major or minor complications, and medications at baseline, were analyzed by Fisher's exact test with correction or the Mann−Whitney test. The historical background of this study was different, leading to differences in the frequency of coronary constriction, provoked spasm and major complications; therefore, we performed propensity score matching analyses. We estimated the propensity score for the maximum ACh 100 μg before August 2012 or maximum ACh 200 μg group using a logistic regression model that included all baseline variables as covariates. Then, we conducted a 1:1 matching of patients between the above two groups with the closest estimated propensity score within a caliper (≤ 0.20 of the pooled SD of estimated logits) using the nearest neighbor method without replacement. *p* < 0.05 was considered significant.

## 3 Results

### 3.1 Clinical Characteristics Among Study Patients

As shown in Figure 1, among 1854 patients with all ACh testing, we excluded 21 patients without intracoronary ACh testing of LCA. Furthermore, we excluded 400 patients without typical or atypical chest pain including 101 patients with cardiomyopathy (hypertrophic cardiomyopathy: 35 & dilated cardiomyopathy: 66) and 299 patients with others (arrhythmia: 88 patients, valvular heart disease: 49 patients, syncope: 22 patients, heart failure: 35 patients, or 105 other patients). Among final study subject of 1433 patients with angina like chest pain as shown in Table 1, ischemic heart disease (IHD) was observed in 1267 patients, including 510 patients with resting angina, 157 patients with effort angina, 130 patients with rest and effort angina, 203 patients with healed myocardial infarction, and 267 patients with previous PCI, whereas atypical chest pain was observed in 166 patients. Organic stenosis (> 75%) was recognized in 287 patients (20%), while 995 patients (69%) had a history of smoking. Hypertension was observed in 683 patients (48%), whereas 726 patients (51%) had dyslipidemia. Diabetes mellitus was found in 375 patients (26%). At baseline, calcium channel blockers (CCBs) and nitrate/nicorandil were found in 800 (56%) and 617 patients (43%), respectively. Angiotensin converting enzyme inhibitors (ACEIs) and angiotensin receptor blockers (ARBs) were recognized in 281 patients (20%), while statins were observed in 311 patients (22%). Beta‐blockers were found in 151 patients (11%) and antiplatelets were recognized in 524 patients (37%).

### 3.2 Comparisons of Clinical Data Between Patients With Total Maximum ACh 100 μg and Those With Maximum ACh 200 μg in the LCA

As shown in Table 1, no difference regarding age and sex was observed between the two maximums ACh doses. The frequency of organic stenosis was significantly higher in patients with total maximum ACh 100 μg than in those with maximum ACh 200 μg, whereas the incidence of hypertension, dyslipidemia, or previous PCI in patients with maximum ACh 200 μg was markedly higher than in those with total maximum ACh 100 μg, whereas the incidence of myocardial infarction in patients with total maximum ACh 100 μg was remarkably higher than in those with maximum ACh 200 μg. Medications at baseline, including CCBs, beta‐blockers, ACEIs/ARBs, and statins, were significantly higher in patients with a maximum ACh of 200 μg than in those with a total maximum ACh 100 μg.

Table 2 shows that the incidence of coronary constriction and usual chest pain was not different between the ACh doses. The frequency of unusual chest pain was remarkably higher in patients with a maximum ACh of 200 μg than in those with total maximum ACh 100 μg, whereas the incidence of no chest pain was markedly higher in patients with total maximum ACh 100 μg than in those with a maximum ACh of 200 μg. The incidence of ST elevation during ACh testing in patients with total maximum ACh 100 μg was significantly higher than in those with a maximum ACh of 200 μg, whereas the frequency of ST depression during ACh testing in patients with total maximum ACh 100 μg was not different from those with a maximum ACh of 200 μg. The frequency of provoked diffuse spasm was not different between the two ACh doses, and the incidence of focal spasm was remarkably higher in patients with total maximum ACh 100 μg than in those with a maximum ACh of 200 μg. The incidence of proximal and mid spasm was significantly higher in patients with total maximum ACh 100 μg than in those with a maximum ACh of 200 μg, while the frequency of distal spasm was remarkably higher in patients with a maximum ACh of 200 μg than in those with total maximum ACh 100 μg. Positive spasm and negative spasm were significantly higher in patients with total maximum ACh 100 μg than in those with a maximum ACh of 200 μg (32% vs. 20%, *p* < 0.001 and 43% vs. 36%, *p* < 0.05), while unclassified results were lower in patients with total maximum ACh 100 μg than in those with a maximum ACh of 200 μg.

**Table 2:** Comparisons of vasoreactivity testing in the left coronary artery between maximum acetylcholine 100 μg and 200 μg in all patients.

	Total maximum ACh 100 μg	*p* value (A vs. B)	(B) Total maximum ACh 200 μg	*p* value (B vs. C)	(C) Maximum ACh 100 μg before August 2012	Maximum ACh 100 μg after August 2012
Number of patients	1234		199		1103	131
Coronary constriction						
Spasm (≥ 90%)	627 (51%)	0.063	87 (44%)	0.440	518 (47%)	109 (83%)***
Chest symptom						
Usual CP	572 (46%)	0.716	95 (48%)	0.215	474 (43%)	98 (75%)***
Unusual CP	38 (3%)	< 0.001	26 (13%)	< 0.001	29 (3%)	9 (7%)*
None	624 (51%)	0.002	78 (39%)	< 0.001	600 (54%)	24 (18%)***
Unknown	0		0		0	0
Ischemic ECG changes						
ST elevation in anterolateral leads	193 (16%)	< 0.001	8 (4%)	< 0.001	160 (15%)	33 (25%)**
ST depression in anterolateral leads	269 (22%)	0.177	55 (28%)	0.013	218 (20%)	51 (39%)***
None	769 (62%)	0.102	136 68%)	0.465	723 66%)	46 (35%)***
Unknown	3 (0.2%)	0.889	0	0.717	2 (0.2%)	1 (1%)
Combination of spasm/usual CP/ischemia						
Spasm & usual CP & positive Ischemia	400 (32%)	< 0.001	39 (20%)	0.005	320 (29%)	80 (61%)***
Spasm & either one positive or none (unclassified results)	298 (24%)	< 0.001	89 (45%)	< 0.001	258 (23%)	40 (31%)
No spasm & no usual CP & no ischemia	536 (43%)	0.044	71 (36%)	0.002	525 (48%)	11 (8%)***
Provoked spasm						
Diffuse spasm	595 (48%)	0.353	103 (52%)	0.044	485 (44%)	110 (84%)***
Focal spasm	253 (21%)	< 0.001	20 (10%)	0.005	198 (18%)	55 (42%)***
Proximal spasm (5/6^a^ & 11^a^)	381 (31%)	< 0.001	33 (17%)	< 0.001	324 (29%)	57 (44%)**
Mid spasm (7^a^)	263 (21%)	0.004	25 (13%)	0.053	203 (18%)	60 (46%)***
Distal spasm (8/9/10^a^ & 12/13/14/15^a^)	204 (17%)	< 0.001	64 (32%)	< 0.001	156 (14%)	48 (37%)***

Abbreviations: ACh, acetylcholine; CP, chest pain.

^a^ Segment,

* *p* < 0.05

** *p* < 0.001

*** *p* < 0.001 versus maximum ACh 100 μg before August 2012.

### 3.3 Comparisons of Clinical Data Between Patients With Maximum ACh 200 μg and Those With Maximum ACh 100 μg Before August 2012 in the LCA

As shown in Tables 1 and 2, the differences between a maximum ACh of 200 μg and a maximum ACh of 100 μg before August 2012 with respect to clinical characteristics and angiographical results during intracoronary ACh tests were approximately the same as the differences between a maximum ACh of 200 μg and total maximum ACh 100 μg. As a different point, Table 2 showed that the frequency of ST depression during ACh tests and diffuse provoked spasms were markedly higher in patients with a maximum ACh of 200 μg than those with a maximum ACh of 100 μg before August 2012. By the physicians' judgments, we could not administer an incremental ACh 200 μg in 22 (7%) of 330 patients who underwent intracoronary ACh vasoreactivity tests after August 2012, although coronary constriction < 90% was obtained at a maximum ACh 100 μg. We could administer intracoronary maximum ACh 200 μg in 199 (90%) of 221 patients who had coronary constriction < 90% at a maximum ACh 100 μg in the LCA.

### 3.4 Spasm Provocation Test Findings in Patients With Variant Angina Showing Anterolateral ST Elevation

As shown in Supporting Information S1: File 1, we summarized the ACh spasm provocation tests of the LCA in 38 patients with variant angina and spontaneous or transient ST elevation in anterolateral leads. Intracoronary injection of ACh 100 μg in the LCA provoked coronary constriction in 25 of 38 patients, whereas two patients revealed coronary constriction by ACh 200 μg but not 100 μg. Furthermore, intracoronary injection of ACh 100 μg did not induce coronary constriction in 10 patients, including one patient under medications, while intracoronary injection of ergonovine (EM) did not provoke coronary constriction in three patients. The sensitivity of maximum ACh 100 μg was 57% (21/37) and it increased up to 62% (23/37) by adding ACh 200 μg in the LCA. Furthermore, we could not obtain more positive case by adding intracoronary EM after ACh testing.

### 3.5 Comparisons of Clinical Characteristics and Vasoreactivity Testing in the LCA Between Maximum ACh 100 μg and 200 μg in Patients With Rest Angina

As shown in Supporting Information S1: File 2, organic stenosis and history of smoking were markedly higher in patients with a maximum ACh of 100 μg, whereas female sex and hypertension were significantly higher in patients with a maximum ACh of 200 μg than in those with a maximum ACh of 100 μg. The incidence of coronary constriction was not different between the two groups. The incidence of unusual chest pain was significantly higher in patients with a maximum ACh of 200 μg than in those with a maximum ACh of 100 μg, while the frequency of usual chest pain was not different between the two groups. Positive spasm and complete negative spasm were significantly lower in patients with a maximum ACh of 200 μg than in those with a maximum ACh of 100 μg (40% vs. 26%, *p* < 0.05, 34% vs. 22%, *p* < 0.05), while unclassified results were markedly higher in patients with a maximum ACh of 200 μg than in those with a maximum ACh of 100 μg (52% vs. 26%, *p* < 0.001). Considering the sensitivity in patients with rest angina, the sensitivity was 40% by a maximum ACh of 100 μg, and it was 26% by a maximum ACh of 200 μg. The frequency of distal provoked spasm was remarkably higher in patients with a maximum ACh of 200 μg than in those with a maximum ACh of 100 μg, whereas the incidence of proximal and mid provoked spasm was significantly lower in patients with a maximum ACh of 200 μg than in those with a maximum ACh of 100 μg. The frequency of diffuse provoked spasm was not different between the two groups, while the incidence of focal provoked spasm was remarkably lower in patients with a maximum ACh of 200 μg than in those with a maximum ACh of 100 μg. Medications at baseline, including CCBs, ACEIs or ARBs, beta‐blockers, and statins, were significantly higher in patients with a maximum ACh of 200 μg than in those with a maximum ACh of 100 μg.

### 3.6 Comparisons of Clinical Characteristics and Vasoreactivity Testing in the LCA Between Maximum ACh 100 μg and 200 μg in Patients With Atypical Chest Pain

Atypical chest pain was observed in 166 patients, including 146 patients with a maximum ACh of 100 μg and 20 with a maximum ACh of 200 μg. Supporting Information S1: File 3 shows that there were no differences regarding clinical characteristics or vasoreactivity testing results between the two ACh doses. The incidence of diabetes mellitus, coronary constriction, the baseline medications, including CCBs, nitrates/nicorandils, ACEIs/ARBs, and statins, were remarkably higher in patients with a maximum ACh of 200 μg than those with a maximum ACh of 100 μg. However, other clinical characteristics and vasoreactivity results were not different between the two groups. Positive spasm was revealed in 7 of 146 patients with a maximum ACh of 100 μg, whereas one patient had coronary constriction, usual chest pain, and ischemic ECG changes in 20 patients with a maximum ACh of 200 μg. Considering the specificity in patients with atypical chest pain, the specificity in patients with a maximum ACh of 100 μg was 95% (139/146), while it was 95% (19/20) in patients with a maximum ACh of 200 μg.

### 3.7 Comparisons of Major and Minor Complications During Intracoronary ACh Testing in the LCA Between Maximum ACh 100 or 200 μg

We show the major and minor complications and procedures in Table 3. Major complications were not different between the maximum ACh 100 μg and maximum ACh 200 μg groups (1.38% vs. 1.51%, *p* = 0.8565). Ventricular fibrillation was observed in each one patient with a maximum ACh of 100 and 200 μg who had necessary electrical defibrillation, while hypotension (< 60 mmHg) was found in two patients with a maximum ACh of 100 μg and one patient with a maximum ACh of 200 μg. Left main trunk equivalent spasm was observed in five patients with a maximum ACh of 100 μg, while intracoronary injection of ACh 200 μg disclosed no left main trunk equivalent spasm. There were no differences regarding major, minor complications or procedures between patients with a maximum ACh of 200 μg and those with a maximum ACh of 100 μg before August 2012. No irreversible complications, including acute myocardial infarction or death, were observed in any of the 1433 procedures of maximum ACh 100 or ACh 200 μg in the LCA. Furthermore, the incidence of paroxysmal atrial fibrillation was not different between patients with a maximum ACh of 100 μg and those with a maximum ACh of 200 μg (1.81% vs. 2.63%, *p* = 0.630).

**Table 3:** Comparisons of major and minor complications during intracoronary acetylcholine testing in the left coronary artery.

	Total	(A) Total maximum ACh 100 μg	*p* value (A vs. B)	(B) Total maximum ACh 200 μg	*p* value (B vs. C)	(C) Maximum ACh 100 μg before August 2012	Maximum ACh 100 μg after August 2012
Number of patients	1433	1234		199		1103	131
Major complications	20 (1.40%)	17 (1.38%)	0.856	3 (1.51%)	0.490	12 (1.09%)	5 (3.82%)*
Non‐sustained ventricular tachycardia	8 (0.56%)	7 (0.57%)	0.689	1 (0.50%)	0.631	5 (0.45%)	2 (1.53%)
Sustained ventricular tachycardia	1 (0.07%)	1 (0.08%)	0.280	0	0.847	1 (0.09%)	0
Ventricular fibrillation	2 (0.14%)	1 (0.08%)	0.642	1 (0.50%)	0.282	1 (0.09%)	0
Hypotension (< 60 mmHg)	3 (0.21%)	2 (0.16%)	0.889	1 (0.50%)	0.152	0	2 (1.53%)*
LMT equivalent spasm	5 (0.35%)	5 (0.41%)	0.802	0	0.514	4 (0.36%)	1 (0.76%)
Cardiac tamponade	1 (0.07%)	1 (0.08%)	0.280	0	0.847	1 (0.09%)	0
Procedures							
Electrical defibrillation	3 (0.21%)	2 (0.16%)	0.889	1 (0.50%)	0.392	2 (0.18%)	0
Cardiac compression	1 (0.07%)	1 (0.08%)	0.280	0	0.847	1 (0.09%)	0
Surgical drainage	1 (0.07%)	1 (0.08%)	0.280	0	0.847	1 (0.09%)	0
Paf or chronic af rhythm before ACH testing	29 (2.02%)	20 (1.62%)	0.013	9 (4.52%)	0.006	15 (1.36%)	5 (3.82%)
Sinus rhythm before ACH testing	1404 (97.98%)	1214 (98.38%)	0.013	190 (95.48%)	0.006	1088 (98.64%)	1088 (98.64%)
Minor complications							
Paroxysmal atrial fibrillation	27 (1.92%)	22 (1.81%)	0.630	5 (2.63%)	0.223	16 (1.47%)	6 (4.76%)***
Administration of anti‐arrhythmic agents	6 (0.43%)	4 (0.33%)	0.410	2 (1.05%)	0.162	3 (0.28%)	1 (0.79%)

Abbreviations: ACh, acetylcholine; af, atrial fibrillation; Paf, paroxysmal atrial fibrillation.

* *p* < 0.05

*** *p* < 0.001 versus maximum ACh 100 μg before August 2012.

### 3.8 Comparisons of the Incidence of Coronary Constriction ≥ 90%, Provoked Spasm, or Major Complications After Propensity Score Matching Between Patients With Maximum ACh 100 μg Before August 2012 and Those With Maximum ACh 200 μg

As shown in Table 4, the incidence of coronary constriction ≥ 90% after propensity score matching was significantly higher in patients with a maximum ACh of 100 μg before August 2012 than those with a maximum ACh of 200 μg, whereas the incidence of provoked spasm was not different after propensity score matching. Furthermore, the incidence of usual chest pain and major complications during ACh vasoreactivity tests were not different after propensity score matching. However, the incidence of unusual chest pain before and after propensity score matching in patients with a maximum ACh of 200 μg was markedly higher than those with a maximum ACh of 100 μg before August 2012.

**Table 4:** Comparisons of clinical characteristics and vasoreactivity testing after propensity score matching in patients with maximum ACh 100 μg before August 2012 and those with maximum ACh 200 μg.

	Maximum ACh 100 μg before August 2012	Maximum ACh 200 μg	*p* value
Number of patients	191	191	
Age (years)	68 ± 9	68 ± 11	0.313
Sex (male/female)	126/65	125/66	0.914
Organic stenosis	12 (6%)	9 (5%)	0.814
Smoking	123 (64%)	122 (64%)	0.915
Dyslipidemia	116 (61%)	111 (58%)	0.836
Diabetes mellitus	70 (37%)	56 (29%)	0.507
Hypertension	121 (63%)	120 (63%)	0.529
Classification of disease (rest AP/effort AP/rest & effort AP/MI/post P/atypical CP)	68 (35%)/22 (12%)/13 (7%)/24 (13%)/42 (22%)/22 (12%)	81 (42%)/18 (9%)/13 (7%)/8 (4%)/51 (27%)/20 (10%)	0.315
IHD	169 (88%)	171 (90%)	0.277
Calcium channel blockers	140 (73%)	141 (74%)	0.819
Nitrates/nicorandils	81 (42%)	82 (43%)	0.604
ACEIs/ARBs	55 (29%)	54 (28%)	0.909
Beta‐blockers	31 (16%)	33 (17%)	0.679
Statins	77 (40%)	71 (37%)	0.402
Antiplatelets	74 (39%)	73 (38%)	0.916
Coronary constriction (spasm [≥ 90%])	84 (44%)	51 (27%)	< 0.001
Spasm (≥ 90%) & usual CP & positive ischemia	51 (27%)	36 (19%)	0.087
Spasm & either one positive or none (unclassified results)	43 (23%)	87 (46%)	< 0.001
No spasm & no usual CP & no ischemia	97 (50%)	68 (36%)	0.003
Usual chest pain	77 (40%)	92 (48%)	0.149
Unusual chest pain	2 (1%)	26 (14%)	< 0.001
Major complications	4 (2.09%)	3 (1.57%)	1.000

Abbreviations: ACEI, angiotensin converting enzyme inhibitor; ACh, acetylcholine; AP, angina pectoris; ARB, angiotensin receptor blocker; CP, chest pain; IHD, ischemic heart disease; MI, myocardial infarction; post P, post percutaneous coronary intervention.

## 4 Discussion

We verified the clinical usefulness and safety of a maximum ACh of 200 μg in the LCA as a spasm provocation test in this article.

### 4.1 Comparisons of Previous Reports

According to previous report by Yasue et al., more than 99% transient stenosis by intracoronary ACh in anterolateral lesions was higher than in the inferior leads (100% [12/12] vs. 76% (16/21), *p* = 0.066) when they performed intracoronary ACh testing in patients with variant angina [1]. They could not reproduce provoked spasm by intracoronary ACh testing in 7 of 70 variant angina patients, including two patients with paroxysmal atrial fibrillation during the manipulation of a catheter in the coronary sinus, one with sinus tachycardia of > 120 beats/min during angiographic study, one after injection of atropine due to sinus bradycardia and hypotension, one with very low disease activity who had no spontaneous attack in the last half year, and two patients with no reasons [2]. Intravenous administration of EM was performed in five of seven patients, and coronary spasm was induced in three patients. The remaining two patients had no provoked spasm by either ACh or EM tests. Cardiologists may perform intracoronary vasoreactivity testing of ACh when they cannot find spontaneous ST changes during chest pain attacks. In patients with low‐moderate disease conditions regarding coronary spasm, cardiologists may fail to diagnose a positive spasm irrespective of performing intracoronary ACh testing.

### 4.2 ACh Testing Was Established as Targeting Variant Angina

Vasoreactivity testing, such as EM and ACh, may be established in patients with variant angina who show ST elevation by spontaneous and/or noninvasive spasm provocation tests [10]. Variant angina may have a very high disease state compared with rest angina patients who demonstrated no ischemic ECG changes during attacks. Cardiologists do not always perform vasoreactivity testing in patients with active variant angina. Vasoreactivity testing is employed in most patients with VSA who have borderline or no ischemic ECG changes during attacks to verify the presence of coronary artery spasm. The sensitivity of standard ACh testing was approximately 90% in patients with variant angina. Therefore, clinical cardiologists may fail to reproduce coronary spasms in some patients with a low‐moderate disease state and VSA. If cardiologists employed higher doses of coronary stimulators to provoke coronary spasm, they may fall in pseud‐positive of intracoronary ACh testing. However, cardiologists should perform ACh 200 μg in the LCA prudently in the cardiac catheterization laboratory when strongly suspected VSA. Physicians should diagnose positive provoked spasm comprehensively by not only angiographical coronary responses by invasive spasm provocation tests but also ischemic ECG changes and usual chest pain during vasoreactivity tests.

### 4.3 Clinical Characteristics of Coronary Responses by ACh 200 μg in the LCA Compared With Maximum ACh 100 μg

The incidence of distal provoked spasm and occurrence of unusual chest pain during ACh testing in patients with maximum ACh 200 μg were significantly higher than that in those with maximum ACh 100 μg, whereas proximal/mid provoked spasm and ST segment elevation during ACh tests in patients with maximum ACh 100 μg was markedly higher than that in those with maximum ACh 200 μg. Ong et al. reported the high incidence of diffuse and distal spasm by intracoronary ACh testing, including ACh 200 μg in the LCA [10]. Our findings were also similar to those of European reports.

### 4.4 Usefulness of ACh 200 μg in the LCA

The injection of ACh 200 μg disclosed coronary constriction ≥ 90% in 44% (87 pts), usual chest pain in 48% (95 pts), ischemic ECG changes in 32% (63 pts) of 199 patients in the LCA. Furthermore, positive spasm with coronary constriction ≥ 90%, usual chest pain, and ischemic ECG changes was recognized in 20% (39 pts) of 199 patients. Coronary constriction was revealed in two variant angina patients with maximum ACh 200 μg but no provoked spasm by ACh 100 μg in the LCA. The sensitivity of ACh in patients with variant angina and ST elevation in the anterolateral leads increased to 71% (27/38) from 66% (25/38). In 166 patients with atypical chest pain, coronary constriction ≥ 90%, usual chest pain or ischemic ECG changes were observed in 25 patients (15%), 31 patients (19%), or 21 patients (13%), respectively. Positive spasm was found in eight patients, including seven patients with a maximum ACh of 100 μg and one patient with a maximum ACh of 200 μg. The specificity of maximum ACh 200 μg was not different from that of maximum ACh 100 μg (95% vs. 95%, *p* = 0.605).

### 4.5 Safety of ACh 200 μg in the LCA

Takagi et al. reported that the incidence of major complications during spasm provocation tests was 6.7%, whereas Aziz et al. reported that the incidence of complications during the ACh test was 3.6% of 1379 patients [11, 12]. The intracoronary ACh injection time was 20 s by Takagi et al., but Aziz et al. administered intracoronary ACh for 3 min. According to our previous review, serious major complications, such as ventricular fibrillation, ventricular tachycardia, bradycardia, cardiogenic shock, cardiac tamponade, acute myocardial infarction, aorta coronary bypass surgery, and death, were 0.95% (148/15 527) [13]. In this article, in patients with ACh testing in the LCA, major complications were observed in 20 patients (1.40%), including 17 patients (1.38%) with a maximum ACh of 100 μg and three patients (1.51%) with a maximum ACh of 200 μg. No difference regarding the incidence of major complications was observed between the maximum ACh of 100 and 200 μg in the same way as a report of Takahashi et al. [14] Electrical defibrillation was necessary for three patients, including one ventricular tachycardia with a maximum ACh of 100 μg and two ventricular fibrillations with each maximum AChs of 100 and 200 μg. Cardiac compression is necessary for one patient with a maximum ACh of 100 μg. The need for surgical cardiac drainage due to tamponade was necessary for one patient with a maximum ACh of 100 μg. However, we had no irreversible complications, such as acute myocardial infarction or death, during the intracoronary LCA ACh testing.

### 4.6 Risk of Pseud‐Positive of ACh 200 μg in the LCA

A high dose of coronary spark may demonstrate excessive coronary constriction compared with daily inducible spasm in some patients. The incidence of unusual chest pain in patients with a maximum ACh of 200 μg was markedly higher than in those with a maximum ACh of 100 μg before (13% vs. 3%, *p* < 0.001) and after (14% vs. 1%, *p* < 0.001) propensity score matching. This may be a clinical diagnostic risk for positive spasm. Vasoreactivity testing is the gold standard for coronary artery spasm. However, cardiologists should decide the judgment of positive spasm carefully after comprehensive manners, including the properties of chest symptoms, the results of noninvasive spasm provocation tests, or the improvement effects of sublingual nitroglycerine. At least, we should define positive spasm: coronary constriction ≥ 90%, usual chest pain, and ischemic ECG changes during ACh testing.

### 4.7 Limitations

Several limitations associated with the present study warrant mention. First, this was a retrospective, single‐center, small‐scale study with a non‐randomized design. Second, the number of patients with variant angina and ST elevation in anterolateral leads as a gold standard was 38 patients alone. Third, the medication at baseline between the two groups was different in some portions. Fourth, the injection dose of maximum ACh 100 or 200 μg was entrusted by the physicians' decisions. However, we could administer maximum ACh 200 μg in 199 (90%) of 221 patients who had coronary constriction < 90% at a maximum ACh of 100 μg after August 2012. Fifth, although all drugs were discontinued for ≥ 24 h before the study, long‐acting calcium channel blockers may have residual effects during ACh vasoreactivity tests. Further research is needed to clarify the usefulness and safety of maximum ACh dose in the LCA.

## 5 Conclusions

We compared the incidence of coronary constriction, usual chest pain, and ischemic ECG changes between maximum ACh 100 and 200 μg in the LCA as ACh testing. We verified the clinical usefulness and safety of a maximum ACh of 200 μg in the LCA as routine spasm provocation testing. If necessary, cardiologists may administer ACh 200 μg when they suspect coronary artery spasm in the LCA without provoked spasm by ACh 100 μg.

## Conflicts of Interest

The authors declare no conflicts of interest.

## Supporting information

Supporting information.

## Data Availability

The data that support the findings of this study are available on request from the corresponding author. The data are not publicly available due to privacy or ethical restrictions.
