# Supplementary material for: Safety and Usefulness of Intracoronary Acetylcholine 200 μg Into the Left Coronary Artery as Vasoreactivity Testing: Comparisons With Intracoronary Acetylcholine Maximum 100 μg
Source: Clin Cardiol. 2024 Oct 2;47(10):e70001. doi: 10.1002/clc.70001 (PMC11445603; doi:10.1002/clc.70001)
Supplement: Supplementary file 1 — Supporting information. [file CLC-47-e70001-s001.doc]

**Supplemantary file 1**

**Spasm provocation tests in patients with variant angina showing anterior and lateral ST elevation**

|  |  |  |  |  |  |  |  |  |  |  |  |  |  |
| --- | --- | --- | --- | --- | --- | --- | --- | --- | --- | --- | --- | --- | --- |
| **No** | **Age** | **Sex** | **Organic stenosis** | **ACh**  **10-100 (μg)** | **Spasm** | **ST changes** | **CP** | **ACh 200 (μg)** | **Spasm** | **ST changes** | **CP** | **ER**  **(μg)** | **Spasm** |
|  |  |  |  |  |  |  |  |  |  |  |  |  |  |
|  |  |  |  |  |  |  |  |  |  |  |  |  |  |
| 1 | 77 | M |  | 20/50/100 | No spasm | (-) | (-) |  |  |  |  |  |  |
| 2 | 73 | M |  | 20/50/100 | No spasm | (-) | (-) |  |  |  |  |  |  |
| 3 | 68 | M |  | 20/50/100 | No spasm | (-) | Usual |  |  |  |  |  |  |
| 4 | 63 | M | #7 (75%) | 20/50/100 | No spasm | (-) | Usual |  |  |  |  |  |  |
| 5 | 61 | M |  | 50/100 | No spasm | (-) | (-) |  |  |  |  |  |  |
| 6 | 61 | M |  | 100 | No spasm | (-) | (-) |  |  |  |  |  |  |
| 7 | 65 | M |  | 50/100 | No spasm | (-) | (-) |  |  |  |  |  |  |
| 8 | 53 | M |  | 50/100 | No spasm | (-) | (-) |  |  |  |  | 64 | No spasm |
| 9 | 67 | M | #7 (75%) | 50/100 | No spasm | (-) | (-) |  |  |  |  | 64 | No spasm |
| 10 | 53 | F |  |  |  |  |  |  |  |  |  | 64 | No spasm under M |
| 11 | 59 | M |  | 20/50/100 | No spasm under M | (-) | (-) |  |  |  |  |  |  |
| 12 | 76 | M |  | 20/50/100 | #6 (d) 13 (f) | (-) | Usual |  |  |  |  |  |  |
| 13 | 74 | M |  | 50 | #9 (d) 13 (t) | (-) | Usual |  |  |  |  |  |  |
| 14 | 38 | M |  | 20/50 | #6 (d) 11 (d) | (-) | Usual |  |  |  |  |  |  |
| 15 | 60 | M | #7 (90%) | 10/20/50 | #7 (d) | (-) | Usual |  |  |  |  |  |  |
| 16 | 71 | M | #7 (75%) | 20 | #7 (t) | ST elevation in ANT | Usual |  |  |  |  |  |  |
| 17 | 67 | M |  | 20/50 | #7 (d) | ST elevation in ANT | Usual |  |  |  |  |  |  |
| 18 | 69 | M | #7 (75%) | 20 | #7 (t) | ST elevation in ANT | Usual |  |  |  |  |  |  |
| 19 | 55 | M |  | 20 | #6 (t) | ST elevation in ANT | Usual |  |  |  |  |  |  |
| 20 | 48 | M | #7 (90%) | 20/50 | #7 (t) | ST elevation in ANT | Usual |  |  |  |  |  |  |
| 21 | 55 | M |  | 20/50/100 | #6 (f) | ST elevation in ANT | Usual |  |  |  |  |  |  |
| 22 | 67 | F | #6 (90%) | 20 | #6 (t) | ST elevation in ANT | Usual |  |  |  |  |  |  |
| 23 | 50 | M |  | 50 | #7 (t) | ST elevation in ANT | Usual |  |  |  |  |  |  |
| 24 | 65 | M | #6 (75%) | 20/50/100 | #6 (d) | ST elevation in ANT | Usual |  |  |  |  |  |  |
| 25 | 62 | M | #6 (75%) | 10/20/50 | #6 (t) | ST elevation in ANT | Usual |  |  |  |  |  |  |
| 26 | 70 | F | #7 (90%) | 20/50 | #7 (t) | ST depression in ANT | Usual |  |  |  |  |  |  |
| 27 | 68 | M |  | 20/50 | #7 (t) | ST elevation in ANT | Usual |  |  |  |  |  |  |
| 28 | 72 | M | #6/#11 (50%) | 10/20 | #6 (d) 11 (d) | ST depression in ANT | Usual |  |  |  |  |  |  |
| 29 | 53 | M |  | 50/100 | #6 (f) | ST elevation in ANT | Usual |  |  |  |  |  |  |
| 30 | 59 | M | #6 (90%) | 20 | #6 (t) 13 (t) | ST elevation in ANT | Usual |  |  |  |  |  |  |
| 31 | 68 | M |  | 20 | #6 (t) 11 (d) | ST elevation in ANT | Usual |  |  |  |  |  |  |
| 32 | 68 | M | #7 (90%) | 20/50 | #7 (d) | ST elevation in ANT | Usual |  |  |  |  |  |  |
| 33 | 60 | M |  | 20/50 | #6 (d) | ST elevation in ANT | Usual |  |  |  |  |  |  |
| 34 | 62 | M |  | 20/50 | #6 (d) 11 (d) | ST depression in ANT | Usual |  |  |  |  |  |  |
| 35 | 56 | M | #7 (90%) | 20 | #6 (f) | ST elevation in ANT | Usual |  |  |  |  |  |  |
| 36 | 66 | M | #7 (90%) | 20/50 | #7 (d) 13 (d) | ST elevation in ANT | Usual |  |  |  |  |  |  |
| 37 | 66 | M |  | 20/50/100 | No spasm | (-) | (-) | 200 | #13(d) | ST depression in LAT | Usual |  |  |
| 38 | 45 | F |  | 20/50/100 | No spasm | (-) | (-) | 200 | #8(d) | ST depression in ANT | Usual |  |  |
|  |  |  |  |  |  |  |  |  |  |  |  |  |  |

(ACh: acetylcholine, ER: ergonovine, CP: chest pain, F: female, M: male, (d): diffuse spasm, (f): focal spasm, (t): total spasm, ANT: anterior, LAT: lateral, #: segment, M: medications)

**Supplemantary file 2**

**Comparisons of vasoreactivity testing in the left coronary artery between maximum acetylcholine 100 μg and 200 μg in patients with rest angina**

|  |  |  |  |
| --- | --- | --- | --- |
|  | **Maximum ACh 100 μg** | **Maximum ACh 200 μg** | **p value** |
|  |  |  |  |
|  |  |  |  |
| Number of patients | 428 | 82 |  |
| Age (y) | 64±11 | 66±12 | 0.460 |
| Female | 77 (18%) | 29 (35%) | 0.001 |
| Organic stenosis | 52 (12%) | 3 (4%) | 0.019 |
| Hypertension | 141 (33%) | 50 (61%) | <0.001 |
| Dyslipidemia | 198 (46%) | 44 (54%) | 0.219 |
| Diabetes mellitus | 91 (21%) | 19 (23%) | 0.700 |
| History of smoking | 338 (79%) | 52 (63%) | 0.004 |
| Coronary constriction (>90%) | 265 (62%) | 49 (60%) | 0.712 |
| Chest symptom |  |  |  |
| Usual CP | 256 (60%) | 48 (59%) | 0.829 |
| Unusual CP | 9 (2%) | 15 (18%) | <0.001 |
| None | 163 (38%) | 19 (23%) | 0.011 |
| Unknown | 0 | 0 |  |
| Ischemic ECG changes |  |  |  |
| ST elevation in anterolateral leads | 87 (20%) | 5 (6%) | 0.001 |
| ST depression in anterolateral leads | 96 (22%) | 26 (32%) | 0.071 |
| None | 243 (57%) | 51 (62%) | 0.362 |
| Unknown | 2 (0.5%) | 0 | 0.730 |
| Combination of spasm/usual CP/ischemia |  |  |  |
| Spasm & usual CP & positive ischemia | 172 (40%) | 21 (26%) | 0.013 |
| Unclassified results | 111 (26%) | 43 (52%) | <0.001 |
| No spasm & no usual CP & no ischemia | 145 (34%) | 18 (22%) | 0.038 |
| Provoked spasm |  |  |  |
| Diffuse spasm | 262 (61%) | 58 (71%) | 0.102 |
| Focal spasm | 111 (26%) | 10 (12%) | 0.006 |
| Proximal spasm (#5/6 & #11) | 194 (45%) | 23 (28%) | 0.004 |
| Mid spasm (#7) | 98 (23%) | 9 (11%) | 0.017 |
| Distal spasm (#8/9/10 & #12/13/14/15) | 81 (19%) | 36 (44%) | <0.001 |
| Medications at baseline |  |  |  |
| Calcium channel blockers | 219 (51%) | 64 (78%) | <0.001 |
| Nitrates or nicorandils | 182 (43%) | 30 (37%) | 0.317 |
| ACEI or ARB | 87 (20%) | 26 (32%) | 0.029 |
| Beta-blockers | 40 (9%) | 14 (17%) | 0.048 |
| Statins | 90 (21%) | 26 (32%) | 0.043 |
| Antiplatelets | 124 (29%) | 28 (34%) | 0.348 |
|  |  |  |  |

(ACh: acetylcholine, CP: chest pain, #: segment, ACEI: angiotensin converting enzyme inhibitor, ARB: angiotensin receptor blocker)

**Supplemantary file 3**

**Comparisons of vasoreactivity testing in the left coronary artery between maximum acetylcholine 100 μg and 200 μg in patients with atypical chest pain**

|  |  |  |  |
| --- | --- | --- | --- |
|  | **Maximum ACh 100 μg** | **Maximum ACh 200 μg** | **p value** |
|  |  |  |  |
|  |  |  |  |
| Number of patients | 146 | 20 |  |
| Age (y) | 65±10 | 65±9 | 0.922 |
| Female | 97 (66%) | 9 (45%) | 0.061 |
| Organic stenosis | 10 (7%) | 1 (5%) | 0.867 |
| Hypertension | 59 (40%) | 12 (60%) | 0.096 |
| Dyslipidemia | 50 (34%) | 10 (50%) | 0.169 |
| Diabetes mellitus | 14 (10%) | 9 (45%) | <0.001 |
| History of smoking | 44 (30%) | 10 (50%) | 0.075 |
| Coronary constriction (>90%) | 17 (12%) | 8 (40%) | <0.001 |
| Chest symptom |  |  |  |
| Usual CP | 25 (17%) | 6 (30%) | 0.165 |
| Unusual CP | 7 (5%) | 0 | 0.683 |
| None | 114 (78%) | 14 (70%) | 0.419 |
| Unknown | 0 | 0 |  |
| Ischemic ECG changes |  |  |  |
| ST elevation in anterolateral leads | 2 (1.4%) | 0 | 0.571 |
| ST depression in anterolateral leads | 17 (12%) | 2 (10%) | 0.874 |
| None | 127 (87%) | 18 (90%) | 0.982 |
| Unknown | 0 | 0 |  |
| Combination of spasm/usual CP/ischemia |  |  |  |
| Spasm & usual CP & positive Ischemia | 7 (5%) | 1 (5%) | 0.605 |
| Unclassified results | 28 (19%) | 5 (25%) | 0.540 |
| No spasm & no usual CP & no ischemia | 111 (76%) | 14 (70%) | 0.557 |
| Provoked spasm |  |  |  |
| Diffuse spasm | 17 (12%) | 2 (10%) | 0.874 |
| Focal spasm | 4 (3%) | 2 (10%) | 0.320 |
| Proximal spasm (#5/6 & #11) | 5 (3%) | 2(10%) | 0.435 |
| Mid spasm (#7) | 12 (8%) | 0 | 0.383 |
| Distal spasm (#8/9/10 & #12/13/14/15) | 4 (3%) | 2 (10%) | 0.320 |
| Medications at baseline |  |  |  |
| Calcium channel blockers | 41 (28%) | 18 (90%) | <0.001 |
| Nitrates or nicorandils | 37 (25%) | 11 (55%) | 0.015 |
| ACEI or ARB | 20 (14%) | 8 (40%) | 0.007 |
| Beta-blockers | 18 (12%) | 4 (20%) | 0.550 |
| Statins | 15 (10%) | 8 (40%) | <0.001 |
| Antiplatelets | 27 (18%) | 7 (35%) | 0.086 |
|  |  |  |  |

(ACh: acetylcholine, CP: chest pain, #: segment, ACEI: angiotensin converting enzyme inhibitor, ARB: angiotensin receptor blocker
